# Peripheral blood inflammatory markers in predicting prognosis in patients with COVID‐19. Some differences with influenza A

**DOI:** 10.1002/jcla.23657

**Published:** 2020-11-22

**Authors:** Yan Zhao, Chao Yu, Wei Ni, Hua Shen, Mengqi Qiu, Youyun Zhao

**Affiliations:** ^1^ Department of Clinical Laboratory Hubei Provincial Hospital of Traditional Chinese Medicine Wuhan China; ^2^ Hubei Academy of Traditional Chinese Medicine Wuhan China

**Keywords:** COVID‐19, inflammatory markers, influenza A, SARS‐CoV‐2

## Abstract

**Background:**

To evaluate the ability of peripheral blood inflammatory markers in predicating the typing of COVID‐19, prognosis, and some differences between COVID‐19 and influenza A patients.

**Methods:**

Clinical data on 285 cases laboratory‐confirmed as SARS‐CoV‐2 infection were obtained from a Wuhan local hospital's electronic medical records according to previously designed standardized data collection forms. Additional 446 Influenza A outpatients’ hematologic data were enrolled for comparison.

**Results:**

NLR, SII, RLR, PLR, HsCRP, and IL‐6 were significant higher and LMR was lower in severe COVID‐19 patients than in mild COVID‐19 patients (*p* < .001). PLR and LMR were lower in the individuals with influenza A than those with COVID‐19 (*p* < .01). COVID‐19 patients with higher levels of NLR, SII, RLR, PLR, HsCRP, and IL‐6 and lower LMR were significantly associated with the severe type. AUC of NLR (0.76) was larger while the specificity of IL‐6 (86%) and sensitivity of HsCRP (89%) were higher than other inflammatory markers in predicating the typing of COVID‐19. PT had obvious correlation with all the inflammatory markers except RPR. NLR showed positive correlations with AST, TP, BUN, CREA, PT, and D‐dimer. Patients with high IL‐6 levels have a relatively worse prognosis (HR = 2.30).

**Conclusion:**

Peripheral blood inflammatory markers reflected the intensity of inflammation and associated with severity of COVID‐19.NLR was more useful to predict severity as well as IL‐6 to predict prognosis of COVID‐19. PLR and LMR were initially found to be higher in SARS‐CoV‐2 virus‐infected group than in influenza A.

## Graphical abstract

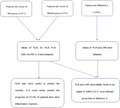

## 1 INTRODUCTION

The epidemic of SARS‐CoV‐2 infection has spread globally, posing a great threat to public health.^1^ SARS‐CoV‐2 infects the human body through the ACE2 receptor and people who was infected has clinical manifestations such as fever, dry cough, fatigue, and respiratory and digestive systems.^2^, ^3^ Patients with mild symptoms account for the majority.^4^, ^5^ The mortality of severe patients with SARS‐CoV‐2 pneumonia is considerable.^6^ Severe patients may develop into septic shock, difficult to correct metabolic acidosis, coagulation dysfunction, and multiple organ dysfunction syndrome (MODS), etc rapidly.^7^ Past studies have confirmed that cytokine storm/systemic inflammatory response syndrome (SIRS) and subsequent compensatory inflammatory response syndrome (CARS) are involved in the pathophysiology of sepsis,^8^ and some studies have found that SARS‐COV‐2‐induced viral sepsis has a large‐scale release of inflammatory cytokines and immunosuppression.^7^, ^9^, ^10^ It has been reported that some new blood inflammatory indexes^11^, ^12^, ^13^ and high‐sensitivity C‐reactive protein (HsCRP),interleukin 6(IL‐6) are related to a variety of inflammatory reactions including sepsis. Recent researches^14^, ^15^ found the disseminated coagulation due to the large production of inflammatory cytokines damaged organs and aggravated the condition. However, further verification is needed in clinical practice. And given the similarity of the symptoms of influenza and COVID‐19, the difference of these inflammatory markers between both patients remains to be found. In this study, we retrospectively analyzed the association of these inflammatory markers with clinical typing and prognosis of COVID‐19 and some differences between patients of influenza and COVID‐19.

## 2 MATERIALS AND METHODS

### 2.1 Data collection

This study included 285 inpatients with COVID‐19 diagnosed at Hubei Provincial Hospital of Traditional Chinese Medicine from January 15, 2020 to February 15, 2020 and 446 outpatients with influenza A from January 1, 2019 to June 1, 2019. The study was approved by the Ethics Committee of Hubei Provincial Hospital of Traditional Chinese Medicine, and all medical records were obtained, including epidemiology, demographics, clinical manifestations, comorbidity, and laboratory data. For inpatients, laboratory data include complete blood count, high‐sensitivity C‐reactive protein (HsCRP), interleukin 6 (IL‐6), alanine aminotransferase (ALT), aspartate aminotransferase (AST), total protein (TP), blood urea nitrogen (BUN), an creatinine (CREA), prothrombin time (PT), and D‐dimer. The laboratory information of outpatients includes complete blood count and HsCRP. The course of disease was defined as the number of days when a patient first noted the onset of symptoms to the day of admission. Comorbidity was determined using the age‐adjusted Charlson Comorbidity Index(aCCI)^16^, ^17^ and was classified into three categories: no comorbidity (aCCI = 0), mild to moderate comorbidity (aCCI = 1–3), and severe comorbidity (aCCI = 4 or more).

### 2.2 Diagnostic criteria

Patients were diagnosed with COVID‐19 according to the "Guidelines for the Diagnosis and Treatment of New Coronavirus Pneumonia" (5th edition) ^18^ issued by the National Health Commission of China. Common patients meet the following conditions: (1) epidemiological history, (2) fever or other respiratory symptoms, (3) abnormal CT images of viral pneumonia, and (4) RT‐PCR positive for SARS‐CoV‐2 RNA result. Severe patients also need to meet any of the following: (1) Respiratory distress, RR ≥ 30 beats/min; (2) In the resting state, pulse oxygen saturation ≤93%; (3) Arterial blood oxygen pressure (PaO2)/inspired oxygen concentration (FiO_2_) ≤300 mm Hg (1 mm Hg = 0.133kPa). The duration of illness was calculated based on the interval between the first appearance of symptoms and the admission examination.

Cases of outpatients recruited for this study during Jan 2019 to Jun 2019 were laboratory‐confirmed with influenza A by real‐time RT‐PCR.

### 2.3 Statistical analysis

Categorical variables are expressed as frequency or percentage, and significance is tested by chi‐square test or Fisher's exact test. The continuous variables of the parameters are expressed as mean ± standard deviation, and significance is tested by *t* test. Non‐parametric variables are expressed as medians and quartiles, and significance was tested by Mann Whitney *U* test. The diagnostic value of selected parameters used to distinguish between mild and severe COVID‐19 patients was evaluated by the receiver operating characteristics (ROC) and the area under the ROC curve (AUC), and the critical value was calculated based on the maximum Youden index. Binary logistic regression analysis was used to select relevant factors that affect patients with mild and severe COVID‐19. Prognostic factors were determined using Cox regression analysis. Analysis was performed using SPSS 24.0 and GraphPad Prism 8 statistical software packages. In all statistical analyses, *p* < .05 was considered statistically significant.

## 3 RESULTS

### 3.1 Demographic and clinical characteristics

Demographic and biochemical characteristics of 285 enrolled patients were summarized in Table 1. All of them were local residents of Wuhan. The cases were 211 mild (74%) and 74 severe (26%), 151 females (53%) and 134 males (47%). There was no significant difference in gender (*p* = .385), the course of the disease was mostly concentrated in 2–3 weeks and has no difference between groups (*p* = .449). Significant difference between two groups was observed in the median age (*p* = .001), aCCI score (*p* < .001), comorbidity categories (*p* < .001) and complications include hypertension (*P* = .002), diabetes (*p* < .001), and heart disease (*p* = .001). Neutrophils (*p* < .001), lymphocytes (*p* < .001), eosinophils (*p* = .001), and the inflammatory markers NLR, SII, RLR, PLR, HsCRP, and IL‐6 were significant higher and LMR was lower in severe patients than in mild (*p* < .001). Our study also showed significantly difference in the AST, TP, BUN, PT, and D‐dimer concentrations (*p* < .05). Different distribution of patients with abnormal ALT and CREA was observed between two groups (*p* < .05), and the prognosis of two groups was also different (*p* < .001) (Table 1).

**TABLE 1:** Clinical characteristics of COVID‐19 patients

	All patients	Mild patients	Severe patients	*p* value
Patients (*n*)	285	211	74	
Gender (*n* (%))	285 (100)	211 (100)	74 (100)	0.385
Male	134 (47)	96 (45)	38 (51)	
Female	151 (53)	115 (55)	36 (49)	
Age (years)	66 (57–70)	64 (56–70)	68 (60–72)	0.001*
Course of disease (*n* (%))				
<7 days	36 (12.6)	25 (11.8)	11 (14.9)	0.449
7–13 days	111 (38.9)	82 (38.9)	29 (39.2)	
14–21 days	117 (41.1)	87 (41.2)	30 (40.5)	
>21 days	21 (7.4)	17 (8.1)	4 (5.4)	
Follow‐up time	5 (3–8)	5 (3–7)	6 (5–8)	<0.001*
Comorbidity (*n* (%))				
Hypertension	16 (5.6)	6 (2.8)	10 (13.5)	0.002*
Diabetes	13 (4.6)	2 (0.9)	11 (14.9)	<0.001*
Heart disease	9 (3.2)	2 (0.9)	7 (9.5)	0.001*
Stroke	11 (3.9)	6 (2.8)	5 (6.8)	0.161
Thyroid disease	1 (0.4)	1 (0.5)	0 (0.0)	1.000
Chronic gastritis	3 (1.1)	2 (0.9)	1 (1.4)	1.000
Hyperuricemia	4 (1.4)	2 (0.9)	2 (2.7)	0.277
aCCI	2 (1–3)	2 (1–3)	3 (1–4)	<0.001*
Comorbidity categories (*n* (%))				
No comorbidity	56 (19.6)	48 (22.7)	8 (10.8)	<0.001*
Mild to moderate comorbidity	183 (64.2)	143 (67.8)	40 (54.1)	
Severe comorbidity	46 (16.1)	20 (9.5)	26 (35.1)	
Complete blood count				
Neutrophil count (×10^9^/L)	3.32 (2.36–5.85)	3.08 (2.27–4.61)	4.54 (2.79–10.59)	<0.001*
Lymphocyte count (×10^9^/L)	1.08 ± 0.46	1.13 ± 0.48	0.96 ± 0.41	<0.001*
Monocyte count (×10^9^/L)	0.36 (0.27–0.53)	0.35 (0.26–0.46)	0.48 (0.27–0.65)	0.204
Eosinophils count (×10^9^/L)	0.04 (0.01–0.07)	0.04 (0.01–0.07)	0.03 (0–0.09)	0.001*
Red blood cell (×10^12^/L)	4.12 ± 0.56	4.12 ± 0.63	4.12 ± 0.32	0.933
RDW (%)	12.3 (11.8–12.8)	12.2 (11.78–12.7)	12.4 (11.88–13.35)	0.942
Platelet count (×10^9^/L)	244 (176–320)	245 (180–320)	239 (162–327)	0.348
Inflammatory markers				
NLR	3.19 (2.00–5.68)	2.89 (1.86–4.88)	4.32 (2.96–10.66)	<0.001*
SII	767 (445–1616)	654 (340–1158)	1032 (594–3364)	<0.001*
RLR	12.14 (9.44–15.35)	11.47 (8.61–15.33)	12.49 (11.25–16.94)	<0.001*
RPR	5.36 (4.17–7.17)	4.92 (4.21–7.08)	5.93 (3.73–8.35)	0.46
PLR	229 (157–311)	229 (153–298)	237 (168–470)	<0.001*
LMR	2.89 (1.77–3.94)	3.13 (2.06–4.15)	1.88 (1.52–3.14)	<0.001*
HsCRP (mg/L)	9.5 (2.1–34.9)	5.1 (1.5–18.4)	24.4 (10.4–86.1)	<0.001*
IL‐6 (pg/ml)	11.36 (4.2–41.26)	8.84 (3.73–24.89)	45.64 (8.05–113)	<0.001*
Coagulation parameters				
PT (s)	12 (11.5–12.7)	11.9 (11.5–12.6)	12.2 (11.7–13.0)	0.008*
D‐dimer (μg/ml)	0.39 (0.26–0.81)	0.36 (0.25–0.68)	0.47 (0.32–1.63)	0.002*
Other laboratory parameters				
ALT (U/L)	20 (16–24)	20 (16–24)	21 (17–26)	0.202
AST (U/L)	33 (18–40)	31 (17–40)	40 (20–63)	0.02*
TP (g/L)	67.8 (63.5–71.9)	67.8 (64–72.5)	67.1 (62.4–69.5)	0.02*
BUN (μmol/L)	4.2 (3.4–5.3)	4.1 (3.4–5.1)	4.6 (3.8–5.8)	0.004*
CREA (μmol/L)	64 (54–79)	6 (54–76)	65 (54–82)	0.24
Abnormal laboratory parameters (*n* (%))				
Abnormal ALT	20 (7)	11 (5.2)	9 (12.2)	0.044*
Abnormal AST	90 (31.6)	60 (28.4)	30 (40.5)	0.054
Abnormal TP	84 (29.7)	62 (29.7)	22 (29.7)	0.992
Abnormal BUN	56 (19.8)	36 (17.1)	20 (27.4)	0.058
Abnormal CREA	39 (13.8)	21 (10.0)	18 (24.7)	0.002*
Prognosis (*n* (%))				
Inpatient	230 (80.7)	210 (99.5)	20 (27.0)	<0.001*
Transferred/ICU	55 (19.3)	1 (0.5)	54 (73.0)	

Note

Data are expressed as *n* (%),mean ± standard deviation, median (interquartile range), as appropriate;Abnormal ALT: male ALT > 40 (U/L), female > 35 (U/L); Abnormal AST: male AST > 50 (U/L), female > 40 (U/L); Abnormal TP: <65 (g/L), Abnormal BUN: male age < 60 years, BUN < 3.1 (μmol/L) or BUN > 8.0 (μmol/L), age > 60 years,BUN < 3.6 (μmol/L) or BUN > 9.5 (μmol/L); female age < 60 years, BUN < 2.6 (μmol/L) or BUN > 7.5 (μmol/L), age > 60 years, BUN < 3.1 (μmol/L) or BUN > 8.8 (μmol/L); Abnormal CREA: male age < 60 years, CREA < 57 (μmol/L) or CREA > 97 (μmol/L), age > 60 years, CREA < 57 (μmol/L) or CREA > 111 (μmol/L); female age < 60 years, CREA < 41 (μmol/L) or CREA > 73 (μmol/L), age > 60 years,CREA < 41 (μmol/L)or CREA > 81 (μmol/L).

* *p* < 0.05.

### 3.2 Some differences between patients with COVID‐19 and influenza A

The general clinical data were compared between 285 COVID‐19 and 446 influenza A patients. There was difference in age, aCCI score, and comorbidity categories between two groups (*p* < .001). After preliminary comparison, PLR and LMR were lower in the individuals with influenza A than those with COVID‐19 (*p* < .01) (Table 2). For the other factors, further expansion of the groups will be necessary.

**TABLE 2:** Clinical characteristics of COVID‐19 patients and influenza A patients

Characteristic	COVID‐19 (*n* = 285)	Influenza A (*n* = 446)	*p* value
Age (y)	63 (51–69)	29 (25–38)	<0.001*
Gender (male%)	134 (47.0)	202 (45.3)	0.648
Comorbidity (*n* (%))			
Hypertension (%)	16 (5.6)	15 (3.4)	0.141
Diabetes (%)	13 (4.6)	10 (2.2)	0.080
Heart disease (%)	9 (3.2)	5 (1.1)	0.05
Stroke (%)	11 (3.9)	8 (1.8)	0.087
Thyroid disease (%)	1 (0.4)	1 (0.2)	1
Chronic gastritis (%)	3 (1.1)	2 (0.4)	0.383
Hyperuricemia (%)	4 (1.4)	4 (0.9)	0.521
aCCI	2 (1–3)	0 (0–0)	<0.001*
Comorbidity categories (*n* (%))			
No comorbidity	56 (19.6)	380 (85.2)	<0.001*
Mild to moderate comorbidity	183 (64.2)	57 (12.8)	
Severe comorbidity	46 (16.1)	9 (2)	
Inflammatory markers			
PLR	229 (157–311)	193 (132–272)	0.004*
LMR	2.89 (1.77–3.94)	1.68 (1.19–2.26)	<0.001*

Note

Data are expressed as *n* (%), median (interquartile range).

* *p* < 0.05.

### 3.3 The ability of inflammatory markers in predicting the type of patients with COVID‐19

We then assessed the correlations between the inflammation markers and the COVID‐19 classification by binary logistic regression analysis. Being highly correlated, however, the variables of inflammatory markers could not be retained in the same model. According to the quartile values of the markers, participants were divided into four groups (Q1–Q4). After adjusting for course of disease, comorbidity categories, abnormal ALT, abnormal CREA, PT and D‐dimer, COVID‐19 patients with higher levels of NLR, SII, RLR, PLR, HsCRP, IL‐6 and lower LMR were significantly associated with the severe type. Compared with patients with low NLR levels, those with high NLR levels were more likely to be severe patients (Adjusted OR = 11.87, 95% CI: 4.02–35.05, *p* < .001) (Table 3).

**TABLE 3:** Binary logistic regression analysis of clinical classification and inflammatory markers of COVID‐19 patients

	Mild patients	Severe patients	Crude OR (95CI)	*p* value	Adjusted OR (95 CI)^a^	*p* value^a^
NLR						
Quartile *n* (%)						
Q1 (<2.0)	65 (30.8)	6 (8.1)	1.0 (reference)		1.0 (reference)	
Q2 (2.0–3.18)	61 (28.9)	10 (13.5)	1.78 (0.61–5.18)	0.293	1.55 (0.50–4.79)	0.445
Q3 (3.19–5.68)	52 (24.6)	20 (27)	4.17 (1.56–11.13)	0.006	3.15 (1.07–9.23)	0.037
Q4 (>5.68)	33 (15.6)	38 (51.4)	12.48 (4.79–32.50)	<0.001	11.87 (4.02–35.05)	<0.001
SII						
Quartile *n* (%)						
Q1 (<445)	63 (29.9)	8 (10.8)	1.0 (reference)		1.0 (reference)	
Q2 (445–766)	60 (28.4)	12 (16.2)	1.58 (0.60–4.12)	0.355	1.12 (0.38–3.28)	0.836
Q3 (767–1616)	53 (25.1)	18 (24.3)	2.68 (1.08–6.64)	0.034	2.44 (0.89–6.65)	0.082
Q4 (>1616)	35 (16.6)	36 (48.6)	8.10 (3.39–19.34)	<0.001	7.04 (2.57–19.28)	<0.001
RLR						
Quartile *n* (%)						
Q1 (<9.44)	66 (31.0)	6 (8.1)	1.0 (reference)		1.0 (reference)	
Q2 (9.44–12.13)	58 (27.6)	13 (17.6)	2.43 (0.87–6.80)	0.091	2.71 (0.92–7.94)	0.069
Q3 (12.14–15.35)	47 (22.4)	26 (35.1)	5.99 (2.29–15.71)	<0.001	4.93 (1.75–13.87)	0.003
Q4 (>15.35)	40 (19.0)	29 (39.2)	7.85 (3.00–20.58)	<0.001	5.07 (1.77–14.54)	0.003
PLR						
Quartile *n* (%)						
Q1 (<157)	71 (33.6)	11 (14.9)	1.0 (reference)		1.0 (reference)	
Q2 (157–228)	57 (27.0)	13 (17.6)	1.45 (0.60–3.48)	0.404	1.37 (0.53–3.51)	0.518
Q3 (229–311)	56 (26.5)	16 (21.6)	1.82 (0.78–4.23)	0.165	1.50 (0.60–3.80)	0.389
Q4 (>311)	27 (12.8)	34 (45.9)	7.78 (3.45–17.56)	<0.001	5.48 (2.22–13.55)	<0.001
LMR						
Quartile *n* (%)						
Q1 (<1.77)	27 (12.7)	29 (39.2)	0.47 (0.23–0.95)	0.035	9.41 (3.40–26.06)	<0.001
Q2 (1.77–2.88)	51 (24.2)	25 (33.8)	0.22 (0.10–0.52)	<0.001	4.14 (1.67–10.24)	0.002
Q3 (2.89–3.94)	46 (21.8)	11 (14.9)	0.10 (0.04–0.23)	<0.001	2.03 (0.73–5.63)	0.172
Q4 (>3.94)	87 (41.2)	9 (12.2)	1.0 (reference)		1.0 (reference)	
HsCRP (mg/L)						
Quartile *n* (%)						
Q1 (<2.1 mg/L)	64 (30.3)	6 (8.1)	1.0 (reference)	<0.001	1.0 (reference)	
Q2 (2.1–9.4mg/L)	64 (30.3)	7 (9.5)	1.17 (0.37–3.66)	0.792	0.99 (0.30–3.30)	0.986
Q3 (9.5–34.9 mg/L)	47 (22.3)	26 (35.1)	5.80 (2.20–15.27)	<0.001	4.30 (1.48–12.51)	0.007
Q4 (>34.9 mg/L)	36 (17.1)	35 (47.3)	10.07 (3.86–26.29)	<0.001	6.91 (2.29–20.89)	0.001
IL–6 (pg/ml)						
Quartile *n* (%)						
Q1 (<4.20)	50 (23.7)	8 (10.8)	1.0 (reference)		1.0 (reference)	
Q2 (4.20–11.35)	57 (27.0)	13 (17.6)	1.47 (0.55–3.91)	0.441	1.20 (0.42–3.48)	0.735
Q3 (11.36–41.26)	52 (24.6)	10 (13.5)	0.98 (0.34–2.82)	0.970	0.90 (0.29–2.76)	0.850
Q4 (>41.26)	52 (24.6)	43 (58.1)	5.38 (2.29–12.63)	<0.001	4.28 (1.70–10.81)	0.002

Note

OR: Odds ratio; CI, confidence interval.

^a^ Indicates adjusted for course of disease, comorbidity categories, abnormal ALT, abnormal CREA, PT, D‐dimer.

**FIGURE 1:**
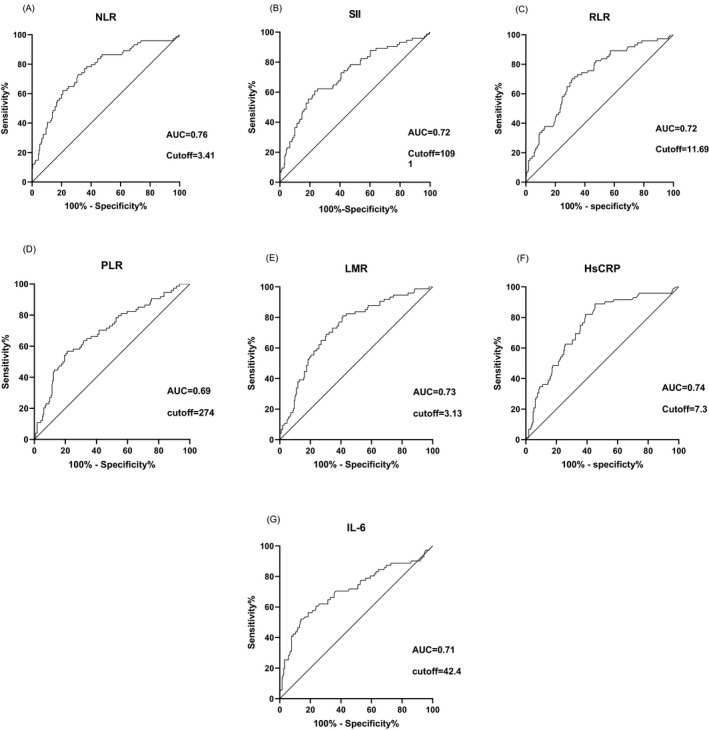
A‐G: Receiver operating characteristic (ROC) curve and Cutoff value of NLR, SII, RLR, PLR, LMR, HsCRP, IL‐6 in differentiating type of COVID‐19

Area under the ROC curve analysis was performed with NLR, SII, RLR, PLR, LMR, HsCRP and IL‐6, and the predictive ability of each index to the clinical classification of patients with COVID‐19 was observed (Figure 1). The results show that compared with SII (0.72), RLR (0.72), PLR (0.69), LMR(0.73), HsCRP (0.74), and IL‐6(0.71), NLR (0.76) has a larger AUC and the predictive ability is superior to the other six inflammatory markers. Cutoff was then calculated based on the ROC curve with the value of NLR 3.41 (specificity: 70%, sensitivity 73%), SII 1091 (specificity: 77%, sensitivity 61%), RLR 11.69 (specificity: 69%, sensitivity 70%), PLR 274 (specificity:79%, sensitivity: 57%), LMR 3.13 (specificity: 81%, sensitivity 58%), HsCRP7.3 (specificity: 55%, sensitivity: 89%), and IL‐6 (specificity: 86%, sensitivity 52%).

### 3.4 Correlation between inflammatory markers and other laboratory parameters

We analyzed the correlation between laboratory indicators includes ALT, AST, TP, BUN, CREA, PT, D‐dimer, and the inflammatory markers respectively. The greatest correlation achieved was by D‐dimer and HsCRP, and the Spearman correlation coefficient was −0.395 (*p* < .001). PT had obvious correlation with all inflammatory markers except RPR,

D‐dimer was also significantly related to inflammatory markers, except for RPR and IL6. Among inflammatory markers, NLR levels were correlated with laboratory indicators (including AST, TP, BUN, CREA, D‐dimer, and PT), and HsCRP was correlated to ALT, AST, BUN, CREA, D‐dimer, and PT. These results showed that there was a clear correlation between coagulation indicators and most inflammatory markers, as well as the correlation between NLR, HsCRP, and disease severity (Table 4).

**TABLE 4:** Correlations between variables

	ALT	AST	TP	BUN	CREA	D‐dimer	PT
NLR							
r	0.074	0.195**	−0.236**	0.258**	0.131*	0.376**	0.323**
*p*	0.228	0.003	<0.001	<0.001	0.028	<0.001	<0.001
SII							
r	0.067	0.186**	−0.180**	0.223**	0.048	0.357**	0.271**
*p*	0.271	0.005	0.003	<0.001	0.419	<0.001	<0.001
RLR							
r	0.119*	0.127	−0.257**	0.202**	0.133*	0.290**	0.239**
*p*	0.049	0.055	<0.001	0.001	0.025	<0.001	<0.001
RPR							
r	0.019	−0.05	−0.046	0.004	0.111	−0.007	0.02
*p*	0.75	0.45	0.454	0.944	0.062	0.909	0.739
PLR							
r	0.103	0.141*	−0.219**	0.187**	0.06	0.288**	0.219**
*p*	0.091	0.033	<0.001	0.002	0.313	<0.001	<0.001
LMR							
r	−0.032	−0.088	0.260**	−0.206**	−0.166**	−0.304**	−0.362**
*p*	0.6	0.181	<0.001	<0.001	0.005	<0.001	<0.001
HsCRP							
r	0.170**	0.237**	−0.072	0.261**	0.256**	0.395**	0.279**
*p*	0.005	<0.001	0.238	<0.001	<0.001	<0.001	<0.001
IL−6							
r	−0.009	0.077	−0.192**	0.096	0.161**	0.072	0.125*
*p*	0.891	0.263	0.002	0.121	0.009	0.244	0.043

* Indicates *p *< 0.05 for the two variables' Spearman correlation.

** Indicates *p *< 0.01 for the two variables' Spearman correlation

### 3.5 Inflammatory markers in predicting the prognosis of patients with COVID‐19

To investigate the associations between the inflammatory markers and the prognosis of COVID‐19, patients were divided into inpatient and transferred/intensive care unit (ICU) group according to prognosis. NLR, SII, RLR, RPR, PLR, LMR, HsCRP, and IL‐6 were included separately in the Cox regression models along with variables that were inconsistent with baseline (including age, course of disease, comorbidity categories, abnormal ALT, abnormal CREA, PT, and D‐dimer). NLR, SII, and IL‐6 were in the final equations. Compared with NLR (HR = 2.07) and SII (HR = 2.00), patients with higher levels of IL‐6 were more likely to have poor prognosis (HR = 2.30) (Table 5).

**TABLE 5:** Results of COX regression models

Covariates	Coefficient	Standard error	*p* value	HR	95%CI	
					Lower	Upper
NLR						
Q1 (<2.0)			0.037			
Q2 (2.0–3.18)	−0.38	0.56	0.492	0.68	0.23	2.04
Q3 (3.19–5.68)	0.14	0.48	0.777	1.14	0.45	2.91
Q4 (>5.68)	0.73	0.43	0.092	2.07	0.89	4.80
SII						
Q1 (<445)			0.017			
Q2 (445–766)	−0.46	0.52	0.379	0.63	0.23	1.75
Q3 (767–1616)	−0.03	0.47	0.943	0.97	0.39	2.42
Q4 (>1616)	0.69	0.41	0.088	2.00	0.90	4.42
IL‐6						
Q1 (<4.20)			0.01			
Q2 (4.20–11.35)	0.37	0.53	0.49	1.44	0.51	4.08
Q3 (11.36–41.26)	−0.63	0.61	0.298	0.53	0.16	1.75
Q4 (>41.26)	0.83	0.45	0.065	2.30	0.95	5.56

Abbreviation: CI, confidence interval; HR, hazard ratio.

## 4 DISCUSSION

Among the baseline data of these two groups of COVID‐19 patients, the median age is higher than other reports^1^, ^6^, ^19^ which may be related to the hospital's own patient source. The previous study has noticed the differences of lymphocytes and neutrophils between mild and sever patients.^10^, ^20^, ^21^, ^22^ Also the higher neutrophils count than healthy people were found in COVID‐19 patients.^23^ In our study, a clear reduction of lymphocytes and increase of neutrophils which was more intense than in mild group were observed in severe patients. The potential reasons of this phenomenon may come from the physiological responses of the innate immune system to systemic inflammation.^24^ It has been reported that ACE2 is the receptor of SARS‐COV‐2 and plays a crucial role in the infection,^25^ lymphocytes which express the ACE2 may be a direct target of viruses that vulnerable to be attacked,^26^ and SARS‐CoV‐2‐induced NKG2A expression may be correlated with functional exhaustion of cytotoxic lymphocytes at the early stage, which may result in disease progression.^27^ Didangelos A^28^ used a computational protein‐protein interaction network to identify possible SARS‐CoV‐2 inflammatory mechanisms and bioactive genes, the study found that neutrophils could be recruited by SARS‐CoV‐2, and lung epithelial cells overexpress neutrophil chemokines after SARS‐CoV‐2 infection. Complement C3 and tumor necrosis factor (TNF) also have been recently shown to be involved in neutrophil activation and prolong neutrophil survival.^22^ Taken together, both lymphopenia and neutrophils increase are the adaptive response of the immune system to SARS‐COV‐2 invasion. NLR was defined as the ratio of neutrophils and lymphocytes; our study found NLR in the severe group was significantly higher than it in the mild; the AUC of NLR is larger than the other four inflammatory markers; binary logistic regression analysis showed that high NLR levels were more likely to be severe patients; and our analysis also shows that NLR was correlated with other laboratory indicators such us AST, TP, BUN, CREA, D‐dimer, and PT. Compared with other inflammatory markers, NLR may well reflect the severity of the immune system affected by SARS‐COV‐2, making it possible to use NLR to identify severe patients.

In contrast to people in mild group, people in sever group were found to have higher IL‐6 levels. IL‐6 also has the greatest specificity in predicting the sever type of patients with COVID‐19 among all the inflammatory markers and is associated with a poor clinical outcome. In the same vein, much of the literature^7^, ^29^, ^30^, ^31^ on COVID‐19 reported the elevated IL‐6 levels which might serve as a predictive biomarker for disease severity.^32^ More evidence suggests that SARS‐CoV‐2 has either immune dysregulation or macrophage‐activation syndrome, both of which are characterized by pro‐inflammatory cytokines,^33^ and the immune dysregulation is driven by the Interleukin‐6 (IL‐6).^34^

Individuals with influenza and COVID‐19 can present with similar symptoms.^35^ Influenza is typical also with the inflammasome in mediating the inflammatory response after infection.^36^, ^37^, ^38^, ^39^ From an epidemiological perspective, the morbidity and mortality of SARS‐CoV‐2 pandemic are much higher than in the pandemic influenza, strongly skewed toward people older than 70 years, and age (<60 years) is the risk factor for severe illness in 1918 and 2009 influenza pandemics, which dissimilar to the SARS‐CoV‐2.^40^ Preliminary comparison of our study showed that PLR and LMR of SARS‐CoV‐2 virus infection are significantly higher than influenza A. For these groups are unbalanced in number (285 vs. 446) and in age (63 vs. 29 years), further expansion of the groups is necessary to other factors and in‐depth comparisons.

Interestingly, not only are PT and D‐dimer observed to be higher in severe patients, they are also significantly associated with inflammatory markers. In accordance with the present results, previous studies^7^, ^14^, ^41^ have demonstrated that higher D‐dimer concentrations associated with poor prognosis. In an observational study,^14^ PT in patients with severe COVID‐19 was shown to be mildly prolonged in patients who died vs. patients who survived. And an anticoagulant therapy^42^ was found to be associated with better prognosis in severe COVID‐19 patients meeting sepsis‐induced coagulopathy (SIC) criteria or with markedly elevated D‐dimer. It is possible that the coagulopathy associated with COVID‐19 is a combination of low‐grade disseminated intravascular coagulation (DIC) and localized pulmonary thrombotic microangiopathy, which could have a substantial impact on organ dysfunction in the most severely affected patients^15^ and could account for some aspects of the results. On the other hand, IL‐6 can induce tissue factor expression on mononuclear cells, which subsequently initiates coagulation activation and thrombin generation. Inflammation‐induced endothelial cell injury also could result in massive release of plasminogen activators. These factors may explain the relatively correlation between the coagulation parameters such as PT and D‐dimer, and the inflammatory markers, and showing how are the immune and coagulation intertwined.

Because of the serious shortage of local medical resources in the early period of the epidemic, the majority patients in this study were not in the early stages of the disease; therefore, the earlier information of inflammatory markers on patient cannot be traced. So, course of disease was used as a covariate in correlation analysis of the inflammation markers and the classification of COVID‐19 patients. Compared with patients with low NLR levels, those with high NLR levels were more likely to be severe patients, which is similar to the findings published by Liu J et al.^43^ The results of COX analysis also revealed that the severe comorbidity and the high levels of NLR were associated with a poor prognosis in patients with COVID‐19, after adjusted for course of disease.

In summary, our study shows that NLR, SII, RLR, PLR, LMR HsCRP, and IL‐6 reflected the intensity of inflammation and associated with severity of patients with COVID‐19. NLR was more useful to predict the severity and IL‐6 could better predict the prognosis of COVID‐19 patients than other inflammatory markers. There was a clear correlation between coagulation indicators and most inflammatory markers. PLR and LMR were initially found to be higher in SARS‐CoV‐2 virus‐infected group than in influenza A.

## CONFLICT OF INTEREST

The authors disclose no conflicts of interest.

## AUTHORS' CONTRIBUTIONS

Yan Zhao, Chao Yu, and Youyun Zhao conceived and designed the experiments and drafted the manuscript. Yan Zhao, Chao Yu, Wei Ni, Hua Shen, and Mengqi Qiu performed the experiments. Yan Zhao and Chao Yu analyzed and interpreted the data.

## Data Availability

The datasets used during the current study are available from the corresponding author on reasonable request.
